# Impact of systemic SARS-CoV-2 vaccination on mucosal IgA responses to subsequent breakthrough infection

**DOI:** 10.1016/j.ebiom.2025.105912

**Published:** 2025-09-16

**Authors:** Ulrika Marking, Oscar Bladh, Katherina Aguilera, Tamas Pongracz, Sebastian Havervall, Nina Greilert-Norin, Kim Blom, Jonas Klingström, Yunzhang Wang, Mikael Åberg, Charlotte Thålin

**Affiliations:** a Department of Clinical Sciences, Karolinska Institutet Danderyd Hospital, Stockholm, Sweden; b Public Health Agency of Sweden, Solna, Sweden; c Department of Biomedical and Clinical Sciences (BKV), Linköping University, Linköping, Sweden; d Department of Medical Sciences, Clinical Chemistry and SciLifeLab Affinity Proteomics, Uppsala University, Uppsala, Sweden

**Keywords:** SARS-CoV-2, Mucosal immunity, Antibody, IgA, Mucosal vaccination

## Abstract

**Background:**

Mucosal IgA responses are central to protection against SARS-CoV-2 infection and viral transmission. While systemic immunity following SARS-CoV-2 infection and vaccination is thoroughly investigated, we have limited understanding of factors affecting the generation and boosting of mucosal IgA.

**Methods:**

In this cohort study, we investigated factors influencing mucosal SARS-CoV-2 IgA responses among 879 healthcare workers enrolled in the longitudinal COMMUNITY study. Blood samples and clinical data were collected from all participants every four months since April 2020. SARS-CoV-2 immune histories are well characterized through national vaccine and infection registries along with regular monitoring of seroconversion of spike and/or nucleocapsid antigen. Regression models were developed to assess the influence of vaccinations and prior infections on the magnitude of SARS-CoV-2 spike-specific IgA in nasal secretions collected from the cohort in October 2022.

**Findings:**

Mucosal SARS-CoV-2 spike-specific IgA was detected in 81% of participants, with a positive association with number of prior infections, indicating a booster effect by reinfection. The increased odds ratio of detectable mucosal IgA remained for at least 22 months post infection. There was a strong association between repeated systemic vaccinations and a lower magnitude of mucosal IgA responses. Moreover, the temporal sequence of infection and vaccination influenced mucosal IgA responses, with higher levels among participants with infection prior to systemic vaccination as compared to those with breakthrough infection as the first viral encounter.

**Interpretation:**

The observation that repeated mucosal exposures elicit enhanced and long-lasting mucosal IgA responses strengthens the rationale for developing effective mucosal vaccines. While systemic vaccination remains essential for preventing severe disease, our findings suggest that it may influence subsequent generation of mucosal IgA trough a reduction of viral load and inflammation in the mucosa. This is highly relevant for both understanding the development of population immunity and for optimizing the timing of a sequential systemic and mucosal vaccination approach.

**Funding:**

This study was supported by grants from Region Stockholm, and 10.13039/501100009252SciLifeLab and the 10.13039/501100004063Knut and Alice Wallenberg Foundation, 10.13039/501100003748SSMF and European Research Council. We thank the Public Health Agency of Sweden for support.

**Research in context**

**Evidence before this study**

A growing body of evidence highlights the critical role of mucosal IgA in protection against SARS-CoV-2 infection, viral replication and onward viral transmission. Previous research has demonstrated that SARS-CoV-2 infection induces mucosal IgA generation, while currently employed systemic vaccines do not appear to elicit a comparable mucosal IgA response. To explore factors influencing the generation of mucosal IgA, we searched PubMed and medRxiv using the terms “mucosal” AND (“IgA” OR “immunity”) AND “SARS-CoV-2”, supplemented by reference lists from relevant publications. While factors related to systemic immune responses following SARS-CoV-2 infection and vaccination are frequently discussed, those influencing the generation of mucosal IgA responses remain less well understood. Despite several reports emphasizing the importance of a mucosal viral encounter, we found no comprehensive investigations addressing the impact of prior immune conferring events on the generation of mucosal IgA.

**Added value of this study**

In this cohort study, we utilized the large COMMUNITY healthcare worker cohort with well-defined SARS-CoV-2 immune histories. Mucosal IgA targeting both ancestral and omicron spike were assessed in nasal secretions collected from 879 regularly anti-nucleocapsid IgG screened participants in October 2022 and the influence of prior SARS-CoV-2 infection and vaccination was assessed by regression models. As expected, our findings indicate a strong relationship between prior infection and mucosal spike IgA levels, with a longer duration than previously shown and a clear mucosal IgA boosting effect by repeated mucosal exposures. However systemic vaccination appeared to limit subsequent generation of mucosal IgA, even when adjusted for the number of and time since prior infections. Moreover, the timing of antigen exposure influenced mucosal IgA generation, with higher levels in those infected before vaccination as compared to those with breakthrough infections. Systemic vaccination remains vital to protect risk groups from severe COVID-19, but these findings provide evidence of an association between systemic vaccination and reduced subsequent generation of mucosal immune responses against SARS-CoV-2 in a large clinical cohort.

**Implications of all the available evidence**

Several vaccines aiming for sterilizing immunity at the respiratory mucosa are under development, some exploring a mucosal boost following systemic priming. Our findings are important for advancing the development of mucosal vaccines, particularly those with replicating platforms, and for understanding the development of population immunity.

## Introduction

Vaccines play a major role in limiting COVID-19-related morbidity and mortality. Replicating virus is however frequently found in the upper respiratory tract of vaccinated individuals,^1^^,^^2^ promoting selection of viral immune evasiveness and the emergence of vaccine resistant strains.^3^ Immunological control of viral transmission likely requires the induction of immunity in the respiratory mucosa, which is not achieved by systemic vaccination.^4^, ^5^, ^6^, ^7^, ^8^, ^9^, ^10^, ^11^ However, despite the crucial role of mucosal IgA in the protection against infection, investigations into humoral immunity have predominantly focused on IgG in blood. This has left a significant void in our understanding of factors affecting the magnitude and duration of mucosal IgA.

Following the rollout of vaccines, it became increasingly clear that the combination of vaccine-induced and infection-induced immune responses—termed hybrid immunity—provides superior protection against SARS-CoV-2 infection and severe disease compared to vaccination alone.^1^^,^^12^ While stronger virus neutralization by serum antibodies may play a role,^13^ this superior protection is likely largely mediated by an effective mucosal immune response at the viral entry point.^1^ Several studies have demonstrated that mucosal IgA exhibit a broader virus neutralizing capacity than serum IgG, not only blocking the virus at the site of entry and thereby reducing the risk of infection but also decreasing onward transmission.^14^, ^15^, ^16^, ^17^ However, although systemic administration of vaccines against respiratory pathogens induces IgG antibodies in serum which may reach the respiratory mucosa via transudation and FcRn-mediated transport, systemic vaccines fail to induce mucosal IgA responses.^10^^,^^11^^,^^18^ While several reports suggest a modest boost in SARS-CoV-2 spike-specific mucosal IgA (hereafter referred to as mucosal spike IgA) levels following systemic SARS-CoV-2 vaccination in COVID-19 convalescents, intramuscularly delivered SARS-CoV-2 vaccines have negligeable effect on mucosal spike IgA responses in individuals naïve to the virus, where no mucosal priming has occurred.^7^^,^^8^^,^^19^^,^^20^ In contrast, mucosal SARS-CoV-2 spike-specific IgG (hereafter referred to as mucosal spike IgG) is readily detected after both primary and booster vaccination, regardless of prior infection. However, although mucosal spike IgG may protect against severe disease by mechanisms of action in the lower respiratory tract,^21^ mucosal spike IgG seem to confer limited long-term protection against infection.^22^ Several attempts to evoke a protective mucosal response by mucosal vaccines are under development,^23^ most of which rely on a combined vaccination regimen with intramuscular priming followed by mucosal boosting. These strategies have been shown to confer protection against infection as well as onward viral transmission in animal models.^24^^,^^25^

Leveraging the longitudinal design of the COMMUNITY healthcare worker cohort, we have previously demonstrated a strong association between high spike-specific IgA levels in the respiratory mucosa and protection against infection for at least eight months, including protection against the omicron sublineages BA.1/BA.2 and BQ.1.^22^^,^^26^^,^^27^ Consistent with other studies, mucosal IgA levels were boosted by re-infections,^22^ but not by a systemic vaccine booster.^10^ While the pivotal role of mucosal IgA in protecting against SARS-CoV-2 infection and viral transmission is well recognized,^4^ factors influencing the titres and duration of mucosal SARS-CoV-2 IgA responses remain to be investigated. In the current study, we again utilized the comprehensive mapping of SARS-CoV-2 immune histories in the COMMUNITY cohort with the objective to explore the impact of vaccinations and prior infections on the magnitude of SARS-CoV-2 spike-specific IgA in nasal secretions from 879 healthcare workers.

## Methods

### Study population

**Fig. 1:**
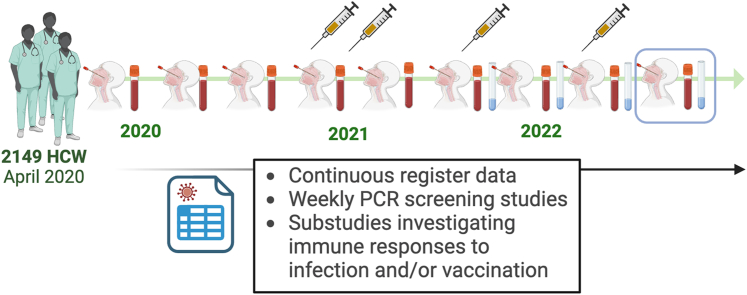
**Study design.** 2149 healthcare workers were enrolled in April 2020. Follow-ups are scheduled every four months and samples are collected from blood and respiratory mucosa. In addition to the regular follow-ups, PCR screening studies were conducted on subsets of the cohort (250–500 participants) during time periods with high viral transmission. Additional information on infections and vaccinations are obtained from health registers. The blue box indicates the samples used in this study.

The ongoing longitudinal COMMUNITY cohort comprises 2149 healthcare workers enrolled at Danderyd Hospital in Stockholm, Sweden in April 2020.^22^^,^^26^^,^^28^, ^29^, ^30^, ^31^, ^32^ Follow-ups are scheduled every four months, with additional sample collections following vaccinations or infections, as well as regular PCR screening programs (weekly self-administered nasal/oropharyngeal/saliva swabs for qPCR for detection of asymptomatic infections) of subgroups (n = 250–500) during periods with high SARS-CoV-2 transmission (Fig. 1). PCR screening studies prior to the samples investigated in this study were conducted December 2020–June 2021, October–December 2021, January–February 2022, June 2022, October 2022.

Follow-up visits every four months include serum and nasal secretion samplings (nasal swabs) and the collection of relevant clinical data (including ongoing immunocompromising disorder or treatment and information on SARS-CoV-2 infections verified through rapid diagnostic tests) through a smart phone-based application. Data on dates and types of vaccination are collected from a Swedish vaccination register (VAL Vaccinera) and information about qPCR-verified SARS-CoV-2 infections are obtained through the national register of communicable diseases, holding all qPCR-positive SARS-CoV-2 cases in Sweden (SmiNet, Public Health Agency of Sweden). Occurrence of SARS-CoV-2 infection between follow-ups is defined as either: 1) seroconversion of serum SARS-CoV-2 spike IgG at any of the study visits prior to vaccination, 2) a positive qPCR test in any of the screening programs, a record of positive qPCR test in the national communicable diseases register (large-scale PCR testing was readily available for healthcare workers within the community testing during the entire study period) or a positive rapid diagnostic test reported at any of the study visits, and/or 3), a rise above cut-off in serum SARS-CoV-2 nucleocapsid-specific IgG between two samplings, or a tenfold increase between two sample time points if already above cut-off.

Primary vaccination regimens were either BNT162b2 (BNT) ×2, ChAdOx1 nCoV-19 (ChAd) ×2 or ChAd + BNT. Booster doses were either BNT or mRNA-1273 (MOD) depending on availability, with transition to their bivalent updated counterparts when available.

To assess factors influencing mucosal spike IgA levels and their SARS-CoV-2 variant cross-binding capacity, serum and nasal secretion samples were analysed from all participants attending the 8th follow-up in October 2022 (n = 1102). To ensure accurate categorization of prior infections, study inclusion was limited to participants that had attended all follow-ups since study enrolment April 2020 (n = 902). Twenty-three participants were excluded from further analyses due to ongoing immunosuppressive drugs or disorders (n = 16) or non-detectable total IgA in the nasal secretion sample indicating incorrect sampling (n = 7, all with detectable ancestral spike IgA in serum), leaving 879 participants for final analyses.

The study was approved by the Swedish Ethical Review Authority (dnr 2020-01653) and conducted in accordance with the Declaration of Helsinki. Written informed consent was obtained from all study participants.

### Mucosal sampling

The soft end of a nasal swab (FLOQSwab cat nr. CP501CS01) was inserted 1–1.5 cm into the nostril, rotated gently, and pressed against the inside of the nostril 4–5 times for a duration of 5 s. The swab was then placed in a sterile 15 mL falcon tube containing 1 mL of phosphate-buffered saline (PBS). The tube was vortexed for 10 s and the swab was pressed against the tube wall to extract as much discharge as possible before being slowly removed. Samples were aliquoted into 50 μl vials and stored at −80 °C until analysis.

### SARS-CoV-2 spike IgG and IgA quantification

Serum (dilution 1:50000) IgG/IgA and nasal secretion (dilution 1:1000) IgA against SARS-CoV-2 ancestral and BA.5 spike were quantified by V-PLEX SARS-CoV-2 panel 31 (Meso Scale Diagnostics, Maryland, USA), and the same panel was used for detection of anti-nucleocapsid IgG in serum. Total IgA concentration in nasal secretions were quantified using Isotyping Panel 1 Human/NHP Kit (Meso Scale Diagnostics) according to the manufacturer’s instructions (dilution 1:5000). To correct for differences in nasal secretion sampling efficacy, ratios between spike IgA > Limit-of-Detection (LoD, average of blank values plus 2.5 standard deviations, set per plate) and total IgA concentrations were calculated for each sample. Ratios are presented multiplied by 10^7^ for graphical purposes.

We have repeatedly demonstrated a strong positive correlation between spike-specific IgA measured by the MSD VPLEX SARS-CoV-2 IgA assay and spike-specific secretory IgA measured by an in-house modification of the same assay in nasal secretions, with Spearman r of >0.9.^10^^,^^26^^,^^33^ The modified assay is described elsewhere.^26^ Briefly, after spike-specific IgA capture and washing, a monoclonal mouse anti-human secretory IgA antibody was used to detect spike-specific secretory IgA (Mouse Anti-Human Secretory IgA, Millipore Cat# 411423-400UG, RRID:AB_10681347 clone HP6141, MerckMillipore).^34^^,^^35^

All plates were analysed on a MESO QuickPlex SQ 120 instrument (Meso Scale Diagnostics). The normalised spike IgA mean + 3SD of fourteen pre-pandemic nasal secretion samples was used to set a cut-off for spike IgA in nasal secretions (3.59 AU/ml for ancestral spike and 4.35 AU/mL for BA.5 spike). Nasal secretion samples with spike IgA < LoD but detectable total IgA were set to 0.1 AU/mL for graphical and statistical purposes.

To determine the SARS-CoV-2 variant cross-binding capacity of mucosal spike IgA, a ratio between BA.5- and ancestral spike binding was calculated for all samples with levels > cut-off in at least one assay (samples with levels < cut-off in one of the assays was set to cut-off/√2 in the division).

### Statistics

Multiple regression models, fitted to different sub-populations according to analyses, were constructed to investigate how SARS-CoV-2 immune histories affect mucosal spike IgA levels (Table 1). First, factors associated with mucosal spike IgA levels above cut-off were explored by a multivariate logistic regression model, estimating effects of age, sex, prior SARS-CoV-2 infection, log_2_-transformed spike serum IgG levels and SARS-CoV-2 vaccination (stratified by 0, 1–3 or >3 received vaccine doses) (Table 1, M1). Next, a similar model was constructed, including time since most recent infection instead of the binary variable prior SARS-CoV-2 infection (Table 1, M2). Time since most recent infection was coded as a categorical variable, with 4 categories (0–7.5 months, 7.5–15 months, 15–22 months and >22 months).

**Table 1:** Overview of regression models and reported outcome of each model.

Model, n (Subgroup)	Dependent variable	Independent variables	Regression type	Estimates reported (Figure illustrating results)
**M1**, 879 (All included participants)	Mucosal spike IgA presence (i.e., IgA levels > cut-off)	Age, sex, prior SARS-CoV-2 infection, log_2_-transformed serum spike IgG levels and SARS-CoV-2 vaccination (stratified by 0, 1–3 or >3 received vaccine doses).	Logistic	Effect of prior infection and SARS-CoV-2 vaccination on OR of mucosal spike IgA presence (Figs. 4A and 5A).
**M2**, 879 (All included participants)	Mucosal spike IgA presence (i.e., IgA levels > cut-off)	Age, sex, time since most recent SARS-CoV-2 infection, log_2_-transformed serum spike IgG levels and SARS-CoV-2 vaccination (stratified by 0, 1–3 or >3 received vaccine doses).	Logistic	Effect of time since most recent infection on OR of mucosal spike IgA presence (Fig. 4A).
**M3**, 706 (All participants with mucosal spike IgA > cut-off)	Log-transformed mucosal spike IgA level	Age, sex, log_2_ transformed serum spike IgG level, number of prior SARS-CoV-2 infections, number of SARS-CoV-2 vaccine doses and time since most recent SARS-CoV-2 infection.	Linear	Effect of number of prior infections and vaccinations on mucosal spike IgA level (Figs. 4B and 5B).
**M4**, 706 (All participants with mucosal spike IgA > cut-off)	Log-transformed mucosal spike IgA level	Age, sex, log_2_ transformed serum spike IgG level, number of prior SARS-CoV-2 infections, infection—vaccination sequence and time since most recent infection.	Linear	Effect of infection—vaccination sequence of mucosal spike IgA level (Fig. 6).
**M5**, 526 (All participants with prior omicron infection and normalized mucosal ancestral *or* BA.5 spike specific IgA > cut-off)	Log-transformed BA.5/ancestral-spike binding ratio	Age, sex, vaccination-infection sequence and time since most recent SARS-CoV-2 infection	Linear	Effect of prior vaccination on the mucosal IgA ancestral/BA.5 spike binding ratio upon omicron infection.

Since mucosal spike IgA levels are linearly associated with protection against SARS-CoV-2 infection,^27^ a linear regression model was constructed to explore factors influencing mucosal spike IgA levels among study participants with levels above cut-off. Log-transformed mucosal spike IgA level was used as dependent variable and age, sex, log_2_-transformed serum spike IgG level, number of prior SARS-CoV-2 infections, number of SARS-CoV-2 vaccine doses, and time since most recent SARS-CoV-2 infection as independent variables (Table 1, M3). A second linear regression model was built to explore the impact of the sequence of SARS-CoV-2 vaccination and infection on mucosal spike IgA levels using the same set of independent variables (Table 1, M4). We found no need for interactions and observed no non-linear associations between mucosal spike IgA levels and serum spike IgG levels or number of past infections and therefore did not apply splines.

Factors associated with a high BA.5/ancestral-spike binding ratio were assessed by two linear regression analyses, one including the full cohort and one including only participants with prior SARS-CoV-2 omicron infection. Ratios were log-transformed to achieve normal distribution. The initial model was constructed with BA.5/ancestral-spike binding ratio as dependent variable and prior infection with ancestral or omicron SARS-CoV-2 as independent variables. Since prior omicron infection was a strong predictor of BA.5/ancestral-spike binding ratio, we next performed a sub analysis on participants with verified omicron infection only, where several independent variables (age, sex, log2-transformed serum spike IgG levels, vaccination-infection sequence, number of vaccine doses and time since most recent infection) were explored in different models.

Likelihood ratio tests were performed and Aikake Information Criterions (AIC) compared to identify the most appropriate set of independent variables, as listed in Table 1. Sensitivity analyses were performed by quantile regression with similar results. All available study participants within the established cohort were included and no a priori power calculations were performed. No adjustments for multiple comparisons were performed. Antibody levels were compared using Mann–Whitney rank sum test. Statistical analyses were performed using GraphPad Prism version 10.4.0 (GraphPad Software, San Diego, California, USA) or The R statistical environment (R version 4.2.2 (2022-10-31), RStudio Team 2019, Boston, USA), using packages tidyverse, glmtoolbox, quantreg, car, lmtest, gvlma.

### Role of funders

None of the funding sources had any role in study design, data analysis, preparation of manuscript or decision to submit the manuscript for publication.

## Results

**Fig. 2:**
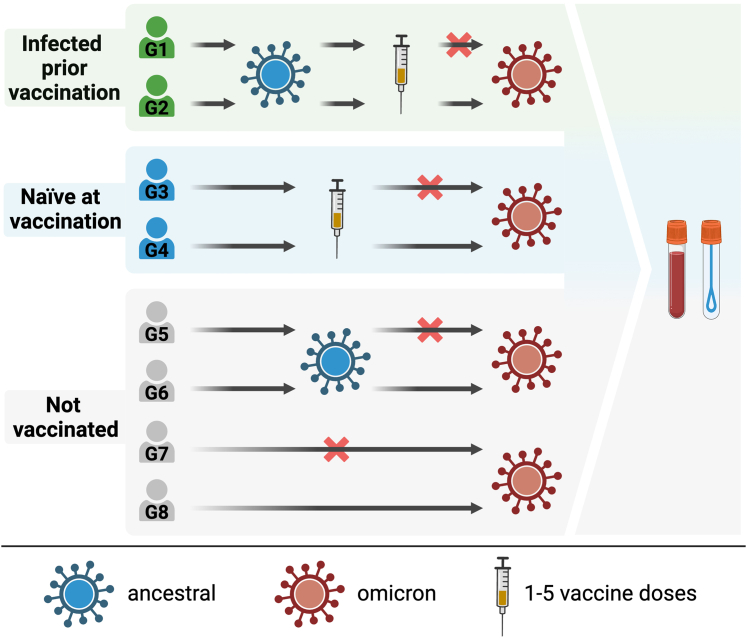
**Schematic overview of the immune histories of study participants**.

The influence of prior SARS-CoV-2 infection and vaccination on mucosal spike IgA was investigated in 879 healthcare workers in October 2022. Participants were stratified based on the number and timing of prior infections and vaccinations (Table 2, Fig. 2, Supplementary Tables S1–S3).

**Table 2:** Demographic characteristics and prior infections and vaccinations in study participants.

	Not infected with omicron (n = 310, 35%)	Infected with omicron (n = 569, 65%)	Overall (n = 879)
**Age**			
Median [Min, Max]	51 [21, 70]	49 [21, 75]	50 [21, 75]
**Sex**			
Female	276 (89%)	501 (88%)	777 (88%)
Male	34 (11%)	68 (12%)	102 (12%)
**Detectable mucosal ancestral spike IgA**			
No	130 (42%)	43 (8%)	173 (20%)
Yes	180 (58%)	526 (92%)	706 (80%)
**Number vaccine doses**			
Not vaccinated	14 (4.5%)	14 (3%)	28 (3%)
1 (not shown)	2 (0.5%)	5 (1%)	7 (1%)
2	16 (5%)	43 (8%)	59 (7%)
3	172 (56%)	355 (62%)	527 (60%)
4	96 (31%)	140 (24%)	236 (27%)
5	10 (3%)	12 (2%)	22 (2%)
**Time since most recent infection**			
No prior infection	107 (35%)	0 (0%)	107 (12%)
0–7.5 months	0 (0%)	373 (66%)	373 (42%)
7.5–15 months	65 (21%)	129 (23%)	194 (22%)
15–22 months	54 (17%)	67 (12%)	121 (14%)
>22 months	84 (27%)	0 (0%)	84 (10%)

At the time of sampling, the majority of participants (89%) had received at least three vaccine doses, 88% had experienced at least one prior infection, and 65% had experienced a prior omicron infection (Table 2). 380 participants (43%) had been infected prior to vaccination (Supplementary Table S4). The median age was 50 years and 88% of the participants were female. Age and sex did not influence mucosal spike IgA levels in any of the models (p > 0.2).

### SARS-CoV-2 infection induces and boosts mucosal spike IgA levels

**Fig. 3:**
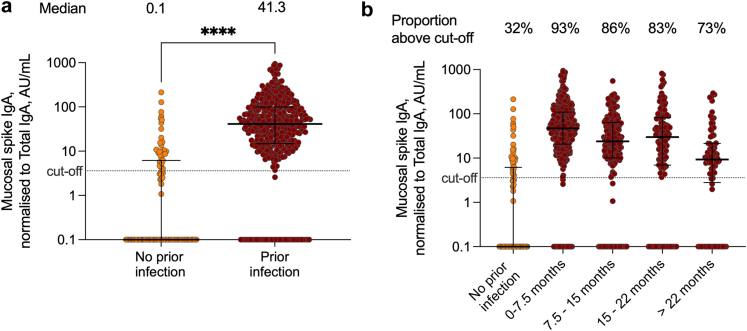
**Mucosal SARS-CoV-2 ancestral spike IgA in all study participants (n = 879), (a) stratified on prior infection and (b) stratified on time since most recent infection. Lines depict median and interquartile range**.

Mucosal spike IgA was detected in the majority of participants (80%; n = 706), with substantially higher levels among participants with prior SARS-CoV-2 infection (median 41.3 [IQR 14.8–99.3] AU/mL) as compared to SARS-CoV-2 naïve participants (0.1 [IQR 0.1–5.93] AU/mL) (Fig. 3).

**Fig. 4:**
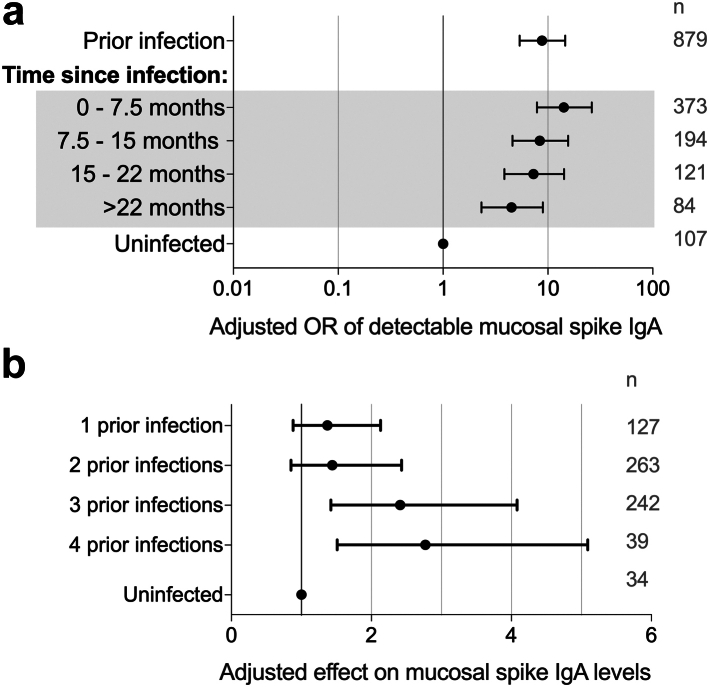
**Influence of prior SARS-CoV-2 infection on mucosal spike IgA. (a)** The effect of prior infection and time since most recent infection on the OR of detectable mucosal spike IgA (multivariate logistic regressions, M1 and M2 in Table 1) **(b)** The boosting effect of repeated infections on mucosal spike IgA levels (multivariate linear regression, M3 in Table 1). OR; Odds Ratio, n; number of participants in each group.

Although mucosal spike IgA levels were found to be lower as time since infection increased, prior infection conferred an increased likelihood of detectable mucosal spike IgA for >22 months post infection (M2, Fig. 4a). Mucosal spike IgA levels increased with increasing number of prior infections, indicating a boost by repeated infections (M3, Fig. 4b).

### SARS-CoV-2 vaccination reduces subsequent generation of mucosal spike IgA

**Fig. 5:**
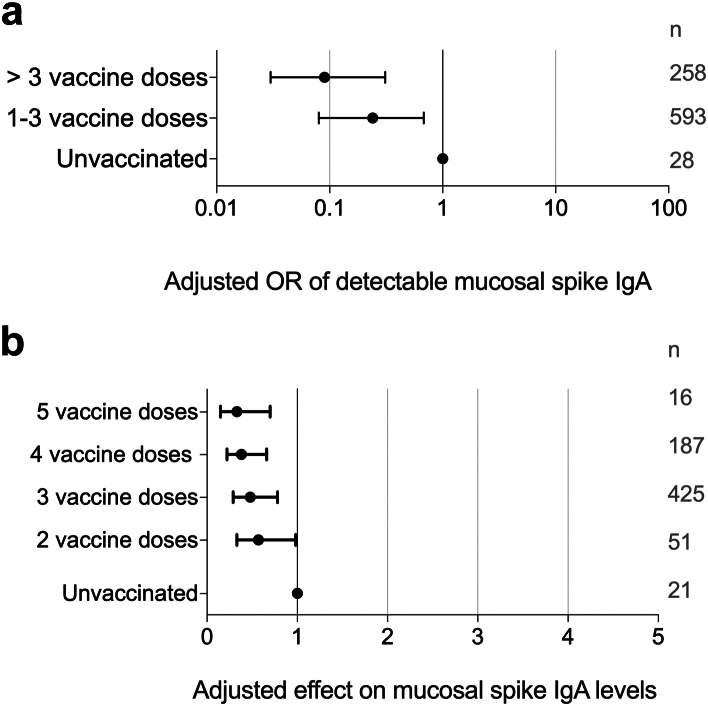
**Influence of systemic vaccinations on mucosal spike IgA. (a)** Effect of vaccination on the OR of detectable mucosal spike IgA (multivariate logistic regression; M1 in Table 1). **(b)** Effect of repeated vaccinations on mucosal spike IgA levels (multivariate linear regression, M3 in Table 1). OR; Odds Ratio, n; number of participants in each group.

Repeated vaccinations strongly influenced the levels of mucosal spike IgA, with an odds ratio (OR) for detectable mucosal spike IgA of 0.24 [95% CI 0.08–0.68] in participants who had received 1–3 vaccine doses, and an OR of 0.09 [95% CI 0.03–0.31] for participants who had received >3 vaccine doses, as compared to unvaccinated participants (M1, Fig. 5a). In line with this, there was an inverse association between the number of vaccine doses and mucosal spike IgA levels (M3, Fig. 5b).

**Fig. 6:**
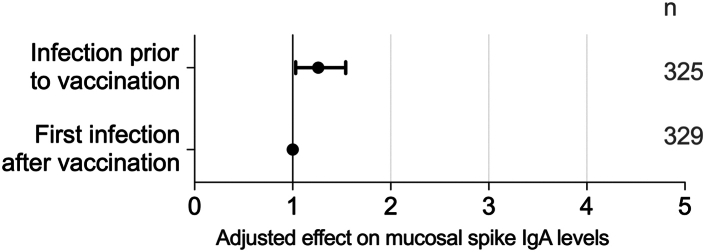
**Influence of the temporal sequence of infection and vaccination on mucosal spike IgA levels, by multivariate linear regression (M4 in**Table 1**)**.

Furthermore, the timing of vaccination relative to infection was also a predictor of mucosal spike IgA levels. Adjusted for time since infection, mucosal spike IgA levels were 1.26-fold [95% CI 1.03–1.54] higher in participants infected prior to vaccination (G1-2 in Fig. 2) as compared to in those in whom the breakthrough infection was the first viral encounter (G4 in Fig. 2) (M4, Fig. 6).

At the time of sampling, SARS-CoV-2 omicron variants had circulated for 10 months in Sweden, with a dominance of BA.1, BA.2 and BA.5.^36^ Omicron variants carry multiple spike mutations with increased transmissibility and partial antibody escape.^37^ We recently demonstrated an enhanced variant cross-binding capacity for mucosal spike IgA compared to serum spike IgG, as indicated by the ratio of BA.5/ancestral-spike binding.^27^ This increased mucosal IgA variant cross-binding capacity furthermore enhanced protection against omicron infection.^27^ We therefore examined the impact of prior infection and vaccination on mucosal IgA BA.5/ancestral spike binding ratio (Supplementary Fig. S1). As expected, participants with prior omicron infection exhibited enhanced binding to BA.5 compared to those without prior omicron infection (1.36-fold higher BA.5/ancestral-spike binding ratio, p < 0.001). This finding prompted us to conduct a sub-analysis on the impact of vaccination within a group restricted to participants with verified omicron infection, however vaccination did not affect the BA.5/ancestral-spike binding ratio significantly (M5).

## Discussion

Mucosal immunity is a crucial actor in the process leading to gradual shift from unconstrained SARS-CoV-2 transmission towards endemic viral control. Protection at the respiratory mucosa relies on the interplay between cellular and humoural immune responses, with mucosal IgA playing a critical role in establishing protective immunity. Despite increasing focus on antibodies at the respiratory mucosa, the influence of preexisting immunity on subsequent generation of mucosal spike IgA remains incompletely understood. In this study, we leveraged a large cohort of healthcare workers with well-defined SARS-CoV-2 immune histories to investigate how prior vaccinations and infections affect the magnitude of mucosal spike IgA.

As expected, mucosal spike IgA levels were substantially higher in participants with prior infection(s), also when adjusted for time since vaccination and number of vaccine doses. This finding is consistent with the functionally distinct nature of the mucosal and systemic immune compartments, with mucosal immune responses heavily relying on localized antigen exposure within the mucosa.^4^

We previously showed that mucosal spike IgA levels remained elevated for up to seven months following omicron infection in triple-vaccinated healthcare workers in our cohort,^26^ and we have demonstrated that also low levels of mucosal spike IgA are associated with protection against omicron BA.1/2 and BQ infections.^26^^,^^27^ Around six to nine months duration of mucosal responses have been repeatedly reported in unvaccinated individuals. Liew et al. observed elevated mucosal spike IgA levels for up to nine months following hospitalization due to COVID-19,^18^ and Fröberg et al. reported elevated mucosal spike IgA levels for up to nine months post infection.^38^ A large study in China, conducted after vaccine roll-out and the initial omicron wave, demonstrated sustained mucosal spike IgA levels for over six months.^39^ The duration of increased protection against reinfection found in individuals with hybrid immunity, however, seems to last longer than the stipulated six to nine months.^12^ Interestingly, our current analyses show that prior infection increases the likelihood of detectable mucosal spike IgA for more than two years post infection, suggesting that mucosal priming may generate a longer lasting mucosal immunity than previously reported. Although these findings may be biased towards participants without reinfection due to robust IgA responses, the observation that mucosal responses can persist, even if only in a subgroup, for as long as >22 months is worth noting. What drives this long-term IgA persistence at low levels remains to be investigated, but recurrent exposures to the virus with subclinical infections during periods with high viral transmission may be a contributing factor, although the risk of such infections would be constant across the examined groups.

Consistent with our previous findings of a stronger and more durable mucosal spike IgA response to reinfection as compared to primary infection,^22^^,^^26^ we now demonstrate progressively higher mucosal spike IgA levels with each subsequent SARS-CoV-2 infection. A boosting of immune responses with repeated infections is not surprising, but highly relevant for the development of population-level immunity and for the design of mucosal vaccines. Although sterilizing immunity at the respiratory mucosa is difficult to achieve when viral loads supersede the neutralizing capacity, a progressively stronger mucosal immunity across populations reduces the susceptibility to emerging variants and likely also influences the extent of viral transmission. Serum spike IgG responses have been shown to be of lower magnitude following reinfections as compared to primary infections with omicron,^22^^,^^32^^,^^40^ which may be a consequence of a mechanism called antibody feedback.^41^ However, protection against reinfection is clearly strengthened after reinfection,^26^^,^^42^ likely reflecting a mucosal boost. Irrespective of mechanism, the observation that repeated mucosal exposures appear to preferentially boost mucosal IgA generation in a manner that is not mirrored by serum spike IgG illustrates the decreasing relevance of serum IgG as the transition to endemic SARS-CoV-2 transmission unfolds.

The induction of mucosal immune responses requires presentation of pathogen-associated epitopes to naïve B and T cells by antigen-presenting cells within the Mucosa-Associated Lymphoid Tissue (MALT).^43^ Once activated, B and T cells migrate to the mucosa, where they execute their effector functions and tissue-resident memory cells are established, which can be rapidly reactivated upon reinfection.^44^ Both initial induction and reactivation therefore rely on viral replication and inflammation at the respiratory mucosa. Interestingly, in this cohort, mucosal spike IgA levels were significantly lower in participants who had received repeated systemic vaccinations as compared to unvaccinated participants, also when adjusted for number of infections and time since infection. Systemic COVID-19 vaccination has been a tremendous success, saving millions of lives. Protecting individuals at risk from severe disease and death remains a priority, and systemic vaccination will continue to be a crucial means of protection. However, breakthrough infections, i.e., infections after vaccination, are associated with less pronounced viral replication at the nasal mucosa, as are high serum spike IgG levels prior to infection.^1^ The inflammation required to create a resident memory B- and T cell response is naturally more restricted when viral replication is lower^45^^,^^46^ and may explain why systemic vaccination appear to have a limiting effect on the magnitude of subsequent mucosal IgA generation. Paradoxically, this could increase long-term susceptibility to infection and, even if infections are mild, contribute to sustained viral circulation in the population. While consequences of these findings do not apply to individuals at risk of severe COVID-19, they are important for understanding population immunity and refining vaccination strategies for non-risk groups. Furthermore, the reasoning is relevant to cases where the mucosal component of a mucosal boost vaccination strategy is replicating, since a strong systemic immunity may limit replication and the mucosal inflammation necessary to generate a robust mucosal response. Four of the 17 mucosal vaccine candidates currently under development are based on replicating platforms.^47^

Notably, the observation that a mucosal viral encounter prior to vaccination renders stronger mucosal IgA responses as opposed to a viral encounter after vaccination are in contrast with those of Mao et al. and Lapuente et al., both demonstrating a stronger mucosal immune response to mucosal vaccination in mice with prior systemic vaccination as compared to in mice without a prior systemic immunization.^24^^,^^48^ However, the mucosal vaccines used in these murine models were employing a non-replicating antigen delivery platform, whereas our data reflects mucosal priming by replicating virus. Further research is necessary to determine whether novel mucosal vaccines are capable of generating mucosal immune responses comparable to those induced by infection. The tolerogenic mucosal environment poses challenges to inducing the desired vaccine response, and adjuvants designed to overcome this tolerance must demonstrate a sufficient safety profile. Nevertheless, potential interferences of systemic priming on subsequent generation of mucosal immune responses may carry weight to the temporal sequence of novel vaccine regimes combining systemic and mucosal vaccine.^24^^,^^25^

Although strengthened by the large study cohort with comprehensive SARS-CoV-2 immune histories, this study has several limitations. Firstly, the study cohort is dominated by female participants of working age, which may challenge generalizability of our findings to the broader population. Secondly, participants with more than 3 vaccine doses are generally older, although our statistical adjustments and normalisation to total IgA likely mitigated the impact of age on the results. Moreover, the large sample size allowed statistical analyses exploring potential confounders, and age and sex were not found to influence the outcome in any of the models. SARS-CoV-2 exposure and potential subclinical infections are furthermore likely increased among healthcare workers which may further limit the generalizability of our findings. Finally, accurate data on prior infections are increasingly difficult to obtain as access to PCR-testing declines. We have made every effort to minimize the number of undetected prior infections by utilizing registry data, self-reported rapid diagnostic test results, regular PCR screenings, and analyses of SARS-CoV-2 anti-nucleocapsid IgG in serum at least every four months since April 2020 in all participants, nonetheless undetected infections cannot be entirely ruled out. The risk of such undetected infections is however equally present among participants with distant infections and the control group without documented infections and would therefore not introduce bias.

It should also be noted that our analyses of sequential immune exposures and their impact on mucosal IgA responses investigate responses to infections caused by different SARS-CoV-2 variants, which may vary in their ability to induce inflammation. However, obtaining an accurate comparison of primary infections caused by different variants is not feasible.

Taken together, our large cohort of healthcare workers, each with well-documented histories of SARS-CoV-2 immune-conferring events, enabled us to uncover multiple associations between prior immunizations and mucosal immune responses with potentially wide-reaching translational and clinical relevance. Our findings imply that repeated mucosal antigen encounters appear to boost mucosal immune responses which is promising in the era of extensive mucosal vaccine development. Although vaccination is crucial in limiting severe outcomes, repeated systemic vaccinations may affect the generation of mucosal immune responses upon subsequent breakthrough infections and thereby possibly influence the development of population immunity and the transition to endemic transmission. Further investigations are warranted to determine the optimal temporal sequence of combined systemic and mucosal vaccine regimens.

## Contributors

U.M. and C.T designed the study. U.M., O.B., K.A, T.P, S.H and C.T. analysed and interpreted the data. U.M. prepared the manuscript’s first draft and figures. K.A., N.G., and S.H. collected data and performed laboratory analysis. U.M and C.T both had full access to and verified the underlying data. Y.W. provided statistical expertise. K.B., J.K., and M.Å. provided constructive suggestions for results and discussion, with valuable input in manuscript completion. All authors participated in the discussion and preparation of final manuscript, which has been read, revised and approved by all authors.

## Data sharing statement

De-identified individual participant data that underlie the results reported in this article (text, tables, figures, and appendices) will be made available to qualified researchers upon reasonable request to the corresponding author (C.T.), subject to approval by the institutional review board and a data use agreement. Data will be available for academic, non-commercial purposes, and requests will be considered for up to 10 years after publication. Supporting materials such as the study protocol and statistical code are available upon request.

## Declaration of interests

The authors declare that they have no known competing financial interests or personal relationships that could have influenced the work reported in this article.
