# Supplementary material for: Impact of systemic SARS-CoV-2 vaccination on mucosal IgA responses to subsequent breakthrough infection
Source: eBioMedicine. 2025 Sep 16;120:105912. doi: 10.1016/j.ebiom.2025.105912 (PMC12466159; doi:10.1016/j.ebiom.2025.105912)
Supplement: Supplementary Fig. S1 and Tables S1–S4 [file mmc1.pdf]

Supplementary material

Systemic SARS-CoV-2 vaccination shapes subsequent generation of mucosal IgA responses – a cohort study

U. Marking[1,2], O. Bladh[1], K. Aguilera[1], T. Pongracz[1], S. Havervall[1], N. Greilert-Norin[1], K Blom[1,2], J Klingström[2,3], Y. Wang[1], M. Åberg[4], C. Thålin[1]

[1] Department of Clinical Sciences, Karolinska Institutet Danderyd Hospital, Stockholm, Sweden

[2] Public Health Agency of Sweden, Solna, Sweden

[3] Department of Biomedical and Clinical Sciences (BKV), Linköping University, Linköping, Sweden

[4] Department of Medical Sciences, Clinical Chemistry and SciLifeLab Affinity Proteomics, Uppsala University, Uppsala, Sweden

Table S1. Mucosal ancestral spike-specific IgA normalised to total IgA, stratified on time since most recent infection.

**Months since most recent infection**	**No documented infection (N=107)**	**0-7.5 months (N=373)**	**7.5 – 15 months (N=194)**	**15-22 months (N=121)**	**> 22 months (N=84)**	**Overall (N=879)**
**Mucosal ancestral spike-specific IgA, normalized to total IgA**						
Mean (SD)	8.67 (26.5)	94.0 (141)	50.8 (70.1)	75.9 (137)	30.0 (59.7)	65.4 (116)
Median [Min, Max]	0.1 [0.1, 213]	46.9 [0.1, 946]	23.9 [0.1, 551]	29.9 [0.1, 814]	9.27 [0.1, 292]	26.4 [0.1, 946]

Table S2. Mucosal ancestral spike-specific IgA normalised to total IgA, stratified on number of vaccine doses

**Number of vaccine doses**	**0 (N=28)**	**1 (N=7)**	**2 (N=59)**	**3 (N=527)**	**4 (N=236)**	**5 (N=22)**	**Overall (N=879)**
**Mucosal ancestral spike-specific IgA, normalized to total IgA**							
Mean (SD)	51.7 (67.7	80.7 (97.8)	60.8 (77.3)	64.3 (121)	70.3 (120)	66.8 (80.7)	65.4 (116)
Median [Min, Max]	33.6 [0.1, 253]	69.3 [0.1, 289]	35.4 [0.1, 385]	23.9 [0.1, 872]	28.6 [0.1, 946]	26.5 [0.1, 247]	26.4 [0.1, 946]

Table S3. Mucosal ancestral spike-specific IgA normalised to total IgA, stratified on temporal sequence of infection and vaccination

**Infection – vaccination sequence**	**Infected prior to vaccination (N=377)**	**Infected after vaccination (N=367)**	**No documented infection (N=107)**	**Not vaccinated (N=28)**	**Overall (N=879)**
**Mucosal ancestral spike-specific IgA, normalized to total IgA**					
Mean (SD)	76.3 (132)	71.5 (111)	6.63 (23.1)	51.7 (67.7)	65.4 (116)
Median [Min, Max]	34.1 [0.1, 946]	32.7 [0.1, 872]	0.1 [0.1, 213]	33.6 [0.1, 253]	26.4 [0.1, 946]

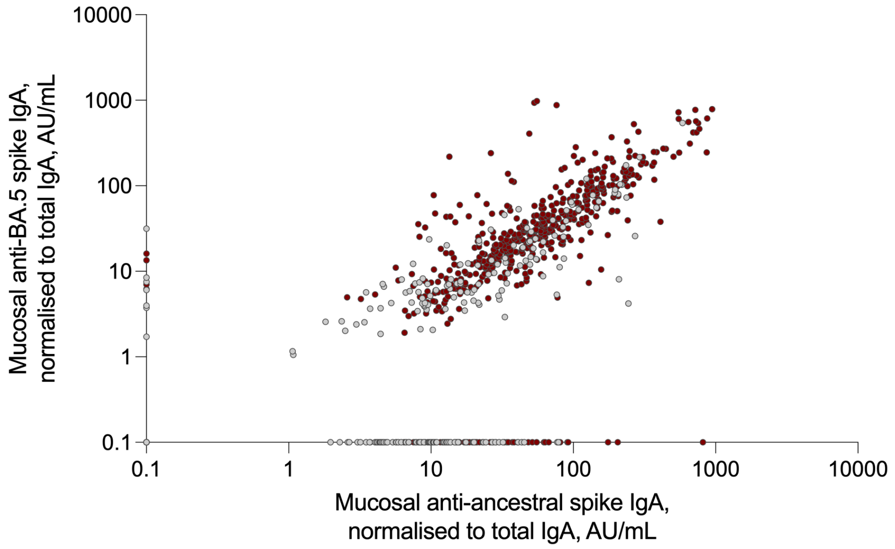

Figure S1. Mucosal IgA binding against ancestral and BA.5 spike. Omicron infected participants are depicted in red.
